# First-line treatments for KRAS-mutant non-small cell lung cancer: current state and future perspectives

**DOI:** 10.1080/15384047.2024.2441499

**Published:** 2024-12-16

**Authors:** Qi He, Xiaoyan Liu, Liyan Jiang, Ping Liu, Weixia Xuan, Yudong Wang, Rui Meng, Huijing Feng, Shuang Lv, Qian Miao, Di Zheng, Yan Xu, Mengzhao Wang

**Affiliations:** a Department of Respiratory and Critical Care Medicine, Peking Union Medical College Hospital, Chinese Academy of Medical Sciences and Peking Union Medical College, Beijing, China; b Department of Respiratory Medicine, Shanghai Chest Hospital, Shanghai Jiao Tong University, Shanghai, China; c Department of Respiratory Medicine, Changsha Hospital Affiliated to Xiangya Medical College, Central South University (The First Hospital of Changsha), Changsha, China; d Department of Respiratory and Critical Care Medicine, Henan Provincial People’s Hospital, Zhengzhou, China; e Department of Medical Oncology, The Fourth Hospital of Hebei Medical University, Shijiazhuang, Hebei Province, China; f Cancer Center, Union Hospital, Tongji Medical College, Huazhong University of Science and Technology, Wuhan, China; g Institute of Radiation Oncology, Union Hospital, Tongji Medical College, Huazhong University of Science and Technology, Wuhan, China; h Department of Thoracic Oncology, Cancer Center, Shanxi Bethune Hospital, Taiyuan, Shanxi, China; i Department of Internal Medicine-Oncology, Inner Mongolia People’s Hospital, Huhehot, Inner Mongolia, P.R. China; j Department of Thoracic Oncology, Clinical Oncology School of Fujian Medical University, Fuzhou, China; k Department of Medical Oncology, Shanghai Pulmonary Hospital, Tongji University, Shanghai, China

**Keywords:** Kirsten rat sarcoma viral oncogene (KRAS), first-line, immune checkpoint inhibitors (ICIs), anti-vascular therapy, chemotherapy

## Abstract

*KRAS* mutations are common in non-small cell lung cancer (NSCLC) and are associated with patient prognosis; however, targeting *KRAS* has faced various difficulties. Currently, immunotherapy, chemotherapy, and chemoimmunotherapy play pivotal roles in the first-line treatment of *KRAS*-mutated NSCLC. Here, we summarize the current evidence on first-line therapies and compare the treatment outcomes and biomarkers for different regimens. KRAS inhibitors and other emerging alternative treatments are also discussed, as combining these drugs with immunotherapy may serve as a promising first-line treatment for *KRAS*-mutated NSCLC in the future. We hope that this review will assist in first-line treatment choices and shed light on the development of novel agents for *KRAS*-mutated NSCLC.

## 1. Introduction

Lung cancer has high morbidity and mortality rates worldwide.^1^ The most common type of lung cancer is non-small cell lung cancer (NSCLC).^2^ Currently, first-line treatment options for unresectable NSCLC without driver mutations include chemotherapy, anti-PD-1/PD-L1 immunotherapy, and chemotherapy combined with immunotherapy.^3^ Advanced NSCLC with driver gene mutations, such as epidermal growth factor receptor (*EGFR*) mutations, are treated with targeted therapies, such as epidermal growth factor receptor tyrosine kinase inhibitors (EGFR-TKIs).^3^

**Figure 1.**
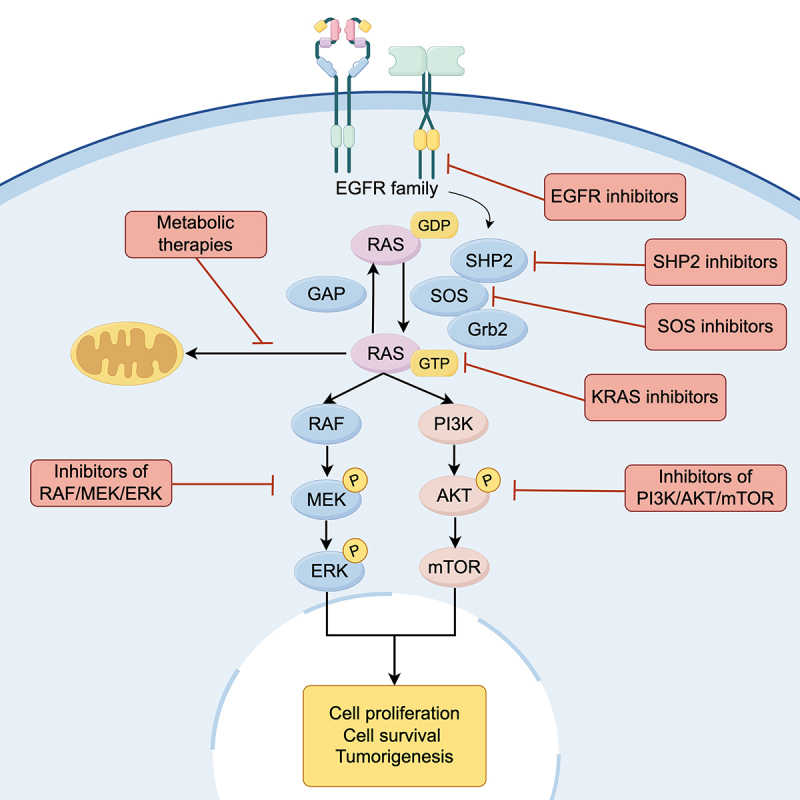
The KRAS signaling pathway.

Kirsten rat sarcoma viral oncogene (*KRAS*) is a common driver oncogene mutation in NSCLC that plays a crucial role in cancer development and evolution.^4,5^ As a member of the GTPase family, it functions to catalyze GTP hydrolysis.^6^ *KRAS* mutations are common in NSCLC; the mutation sites include amino acids 12, 13, and 61, with the G12C mutation being the most common, followed by G12V, G12D, and G12A mutations.^7,8^ *KRAS* mutations reduce GTPase activity, leading to sustained KRAS activation and increased signaling in the downstream pathways. The mitogen-activated protein kinase (MAPK) and phosphatidylinositol-3-kinase (PI3K)-protein kinase B (AKT) pathways are important downstream pathways of RAS signaling (Figure 1). The MAPK cascade plays an important role in cell proliferation and tumorigenesis.^9^ On the other hand, RAS also binds to and activates PI3K, leading to AKT phosphorylation and stimulation of the mammalian target of the rapamycin (mTOR) pathway.^10^ This contributes to cell proliferation and survival, which are essential for tumor development and maintenance.^10^ However, the efficacy and safety of the currently available drugs targeting KRAS remain unsatisfactory.^11^ Therefore, chemotherapy and immunotherapy still play important roles in the first-line treatment of NSCLC with *KRAS* mutations (*KRAS*m).

Although numerous studies have focused on chemotherapy and immunotherapy for *KRAS*m NSCLC, the results from previous studies are inconsistent owing to the heterogeneity of *KRAS*m NSCLC. This review focuses on the best options for the first-line treatment of *KRAS*m NSCLC by reviewing the current evidence from clinical studies.

## 2. Chemotherapy is the cornerstone of first-line treatment for KRASm NSCLC

First-line chemotherapy for *KRAS*m NSCLC resulted in worse treatment outcomes than that for *KRAS*wt NSCLC (Table 1). A retrospective study by Metro et al. showed that patients with *KRAS*m NSCLC (*n* = 77) treated with first-line platinum-containing chemotherapy had a significantly lower objective response rate (ORR), disease control rate, progression-free survival (PFS), and overall survival (OS) than the *KRAS*wt group (*n* = 127).^12^ Similarly, in a single-center retrospective study, Hames et al. compared the outcomes of patients with *KRAS*m (*n* = 80) and driver gene-negative (*n* = 70) advanced NSCLC treated with first-line platinum-containing chemotherapy.^13^ The median PFS in the *KRAS*m group was shorter than that of the driver gene-negative group by 1.2 months (4.5 vs. 5.7 months, *p* = .008), and the median OS was 4.7 months shorter (8.8 vs 13.5 months, *p* = .038), with subgroup analyses for adenocarcinoma and metastatic disease suggesting similar results.^13^ Eklund et al. also demonstrated that the OS of 104 patients with stage IV *KRAS*m NSCLC treated with first-line chemotherapy was significantly shorter than that of 91 patients with *KRAS*wt NSCLC (9 vs. 11 months, *p* = .018). Furthermore, the multivariate Cox analysis showed that *the KRAS* mutation was a risk factor for shorter OS (hazard ratio (HR): 1.564, *p* = .008).^14^ However, a retrospective study by Mellema et al. showed that the ORR of *KRAS*m NSCLC (*n* = 60) for first-line platinum-containing chemotherapy was similar to that of *KRAS*wt NSCLC (*n* = 101), with a median PFS (4.0 vs. 4.7 months, *p* = .12) and median OS (7.0 vs 9.3 months, *p* = .25) numerically reduced compared to *KRAS*wt NSCLC, without statistical significance.^15^

**Table 1.** First-line treatments for KRAS mutant NSCLC.

References	First author	Year	Treatment regimen	Study design	Number of patients		ORR, %			Median PFS, months			Median OS, months		
					KRASm	KRASwt	KRASm	KRASwt	P value	KRASm	KRASwt	P value	KRASm	KRASwt	P value
^12^	Metro	2014	Platinum-based chemotherapy	Retro	77	127	27.3	42.5	0.04	5.4	6.8	0.05	14.7	23.4	0.21
^13^	Hames	2016	Platinum-based chemotherapy	Retro	80	70				4.5	5.7	0.008	8.8	13.5	0.038
^14^	Eklund	2022	Platinum-based chemotherapy	Retro	104	91							9	11	0.018
			Pembrolizumab	Retro	20	17							23	6	0.006
^15^	Mellema	2013	Platinum-based chemotherapy	Retro	60	101	16.7	21.8		4	4.7	0.12	7	9.3	0.25
^16^	Ghimessy	2019	Platinum-based chemotherapy + bevacizumab	Retro	95	152				7.03	8.63	0.0255	14.23	21.57	0.0186
			Platinum-based chemotherapy	Retro	75	179							10	11	0.6771
^17^	Peters	2017	Atezolizumab	Trial	33	67	27	16		8.4	4.8		NE	20.1	
^18^	Liu	2023	ICI + (chemotherapy)	Retro	20	49				10.1	9	<0.05			
^19^	Sun	2021	ICI monotherapy	Retro	363	342							21.1	13.6	0.03
			ICI + chemotherapy	Retro	210	212							20	19.3	0.93
^20^	Frost	2021	Pembrolizumab	Retro	62	57	50.9	46	0.62	13.3	6.2	0.05	23.4	26.1	0.74
^21^	Li	2022	ICI + chemotherapy	Retro	23	57				12.8	9.7	<0.001	21	15.9	0.01
^22^	Justeau	2022	Pembrolizumab	Retro	G12C vs non-G12C: 86 vs 141)	454	47 vs 40	45		7 vs 4.8	8.5	0.2284	18.4 vs 20.6	27.1	0.5664
^23^	Noordhof	2021	Pembrolizumab	Retro	338	257							19.2	16.8	0.86
^24^	Kartolo	2021	ICI monotherapy	Retro	30	29	37	26	0.268	6	5.4	0.416	12.9	19.3	0.87
^25^	Gadgeel	2019	Pembrolizumab + chemotherapy	Trial	59	145	40.7	47.6		9	9		21	23	
			Chemotherapy	Trial	30	55	26.7	10.9		5	5		14	9	
^26^	Nakajima	2022	ICI + chemotherapy	Pool	219	313	46	51					22.4	18.7	
			ICI monotherapy	Pool	135	240	37	33					16.2	16.4	
			Chemotherapy	Pool	201	322	35	32					17.1	14.9	
^27^	Alessi	2023	ICI + chemotherapy	Retro	351	526	33	39		5.7	5.9	0.21	14.1	15	0.42
^28^	Veccia	2023	ICI + (chemotherapy)	Retro	50	69							14.7	14.9	0.529
^29^	Mok	2023	Pembrolizumab	Retro	30	127	56.7	29.1		12.3	5.8		28.4	14.8	
			Platinum-based chemotherapy	Retro	39	105	18	21		6.2	6.3		11	12.1	
^30^	Liu	2022	ICI + (chemotherapy)	Retro	50		44			11.7			23.8		
			Chemotherapy + (AT)	Retro	115		30.43			7			14.7		
^31^	Gu	2023	ICI + (chemotherapy)	Retro	33		27.2			7.4			24.1		
			Chemotherapy	Retro	37		16.2			4.5			13.2		
^32^	Wang	2023	ICI + chemotherapy	Retro	11	29				10.6	13.3		24.6	NR	
			Chemotherapy + (AT)	Retro	10	23				7.2	6.9		13.3	21.1	
^33^	Sun	2022	ICI + chemotherapy	Retro	76		47.4			16.9			37.1		
			Chemotherapy	Retro	74		31.1			4.6			19.8		
			Chemotherapy + AT	Retro	33		21.2			7			20.7		
^34^	West	2022	Atezolizumab, bevacizumab, carboplatin, paclitaxel(ABCP)	Trial	80	235	8.4			8.1			19.8	18.9	
			Atezolizumab, carboplatin, paclitaxel (ACP)	Trial	74	234	6.8			4.8			11.7	19.5	
			Bevacizumab, carboplatin, paclitaxel (BCP)	Trial	71	226	7			5.8			9.9	18.2	

*ORR(objective response rate), PFS(progression-free survival), OS(overall survival), ICI(immune checkpoint inhibitors), AT(antivascular therapy), KRASm(KRAS mutant), KRASwt(KRAS wild-type), Retro(retrospective), Trial(clinical trial analysis), Pool(pooled analysis), NR(not reach).

As a commonly used agent for non-squamous NSCLC, the reported efficacy of pemetrexed in *KRAS*m NSCLC varies widely between studies. Several studies have suggested the survival benefit of pemetrexed-containing chemotherapy. A retrospective analysis of 115 patients with *KRAS*m NSCLC treated with first-line chemotherapy by Liu et al. demonstrated that pemetrexed-containing regimens (*n* = 60) were associated with a longer PFS (10.1 vs 6.2 months, *p* < .001) and OS (16.4 vs 14.1 months, *p* = .112), although the OS benefit was not statistically significant.^30^ Similarly, Chen et al. compared the outcomes of three first-line chemotherapy regimens, pemetrexed/platinum (PP, *n* = 198), gemcitabine/platinum (GP, *n* = 64), and paclitaxel/platinum (TP, *n* = 38), in *KRAS*m NSCLC.^35^ Although there was no significant difference in ORR and disease control rate (DCR) among the three regimens, in terms of PFS, the PP group (6.4 months) was significantly prolonged compared to the GP group (4.9 months, *p* = .033) and the TP group (5.6 months, *p* = .05); in terms of OS, the GP group (17.5 months) was significantly prolonged compared to the PP group (24.6 months, *p* = .03) and TP group (26.8 months, *p* < .001). As *KRAS* mutations are predominantly found in non-squamous NSCLC and pemetrexed is more effective in non-squamous cancers, pemetrexed-based chemotherapy regimens are more effective in treating *KRAS*m NSCLC.^36^

However, a retrospective study by Ricciuti et al., which did not differentiate between the number of lines of treatment, suggested that treatment outcomes were worse with pemetrexed-containing chemotherapy regimens for advanced NSCLC.^37^ PP-based regimens (*n* = 81) were associated with a worse ORR (30.9% vs. 47.4%, *p* = .05), DCR (51.8% vs. 71.9%, *p* = .02), PFS (4.1 vs. 7.1 months, *p* = .03), and OS (9.7 vs. 26.9 months, *p* = .002) than non-PP-based regimens (*n* = 57).^37^

Therefore, as the cornerstone of first-line treatment, chemotherapy is less effective in *KRAS*m-advanced NSCLC than in *KRAS*wt NSCLC, and pemetrexed-based chemotherapy regimens may result in a better prognosis.

## 3. Chemotherapy combined with antivascular therapy prolongs survival in the first-line treatment of KRASm NSCLC

*KRAS* mutations are associated with overexpression of VEGF, which is involved in tumor angiogenesis and promotes lung cancer development and metastasis.^38,39^ Antivascular therapy inhibits the process of tumor angiogenesis by inhibiting the binding of VEGF to its receptor. Therefore, chemotherapy combined with antivascular therapy has the potential to serve as a first-line treatment for *KRAS*m NSCLC.

Even with first-line chemotherapy combined with antivascular therapy, patients with *KRAS*m NSCLC still had poorer treatment outcomes than those with *KRAS*wt NSCLC. A study by Ghimessy et al. suggested that compared with *KRAS*wt (*n* = 152), advanced lung adenocarcinoma patients with KRASm (*n* = 95) treated with first-line chemotherapy combined with bevacizumab had a worse OS (14.23 vs. 21.57 months, *p* = .0255) and PFS (7.03 vs 8.63 months, *p* = .0186).^16^ The association between *KRAS* mutations and OS was independent of age, sex, smoking status, Eastern Cooperative Oncology Group performance status (ECOG PS), and tumor stage.^16^

First-line chemotherapy combined with antivascular therapy improved survival in patients with *KRAS*m NSCLC. A single-center retrospective study by Liu et al. demonstrated that first-line chemotherapy combined with antivascular therapy (*n* = 58) significantly prolonged PFS (10.0 vs. 6.5 months, *p* = .031) and OS (19.7 vs. 13.7 months, *p* = .004).^30^ Similarly, a retrospective study by Ghimessy et al. on advanced *KRAS*-mutant lung adenocarcinoma in stages IIIB – IV demonstrated that first-line platinum-containing chemotherapy in combination with bevacizumab (*n* = 95) had a significant OS benefit compared to platinum-containing chemotherapy alone (*n* = 75) (14.23 vs. 10 months, *p* = .0002).^16^

Regarding specific drug selection, Liu et al. showed that among 58 patients treated with chemotherapy combined with antivascular therapy, the ORR in the paclitaxel combined with antivascular drug group (59.09% vs. 30.56% vs. 12.5% vs. 26.92%, *p* = .032, *p* = .001, and *p* = .024) was significantly higher than that in the pemetrexed combined with antivascular drug group, the pemetrexed group, paclitaxel group, while PFS (14.0 vs. 4.0 vs. 8.0 vs. 5.0 months, *p* = .009, *p* = .008, and *p* < .001) and OS (25.0 vs. 10.0 vs. 19.0 vs. 11.0 months, *p* = .006, *p* = .508, and *p* < .001) in the pemetrexed-combined antivascular group were significantly longer than those in the pemetrexed group, the paclitaxel combined antivascular drug group, and the paclitaxel group.^30^ Similarly, a study by Mellema et al. showed that paclitaxel combined with antivascular therapy had the highest ORR (62%, *n* = 38) than paclitaxel (50%, *n* = 30), pemetrexed (21%, *n* = 334), and gemcitabine (25%, *n* = 62).^40^

Thus, first-line chemotherapy combined with antivascular therapy remains significantly less effective in patients with *KRAS*m NSCLC than in those with *KRAS*wt NSCLC but does improve survival in patients with *KRAS*m NSCLC.

## 4. Immunotherapy is the mainstay of first-line treatment for KRASm NSCLC

### 4.1. The efficacy of immunotherapy in patients with KRASm NSCLC is not inferior to those with KRASwt NSCLC

Unlike chemotherapy or chemotherapy combined with antivascular therapy, the efficacy of first-line immunotherapy may be superior in *KRAS*m NSCLC compared to *KRAS*wt (Table 1). A meta-analysis integrating three trials (IMpower-150, Keynote-189, and Keynote-042) demonstrated that *KRAS*m NSCLC had better survival with first-line immunotherapy than *KRAS*wt NSCLC (χ^2^ = 6.26, *p* = .01).^41^ In the BIRCH trial, advanced NSCLC with PD-L1 expression ≥5% in tumor cells or tumor-infiltrating immune cells was treated with first-line atezolizumab in 33 patients that were *KRAS*m patients and 67 patients that were *KRAS*wt.^17^ The ORR (27% vs. 16%), PFS (8.4 vs. 4.8 months), and OS (NE vs. 20.1 months) were higher in patients who were *KRAS*m than in those who were *KRAS*wt.^17^ Similarly, a retrospective study by Liu et al. suggested that 20 patients with *KRAS*m NSCLC treated with first-line anti-PD-1/PD-L1 therapy had a significantly longer PFS than 49 patients with *KRAS*wt NSCLC.^18^ Sun et al. demonstrated that OS (21.1 vs. 13.6 months, *p* = .03) was significantly longer in *KRAS*m NSCLC (*n* = 363) than in *KRAS*wt NSCLC (*n* = 342) with first-line immunological monotherapy, and that the association between *KRAS* mutation status and OS remained significant in the multivariate Cox model (HR = 0.77).^19^ In addition, a study by Eklund et al. showed that stage IV NSCLC with *KRAS*m (*n* = 20) had a significant OS (23 vs. 6 months, *p* = .006) benefit from first-line immunotherapy compared to *KRAS*wt (*n* = 17) and that *KRAS* mutation was a favorable prognostic factor for OS in a multifactorial Cox regression (HR = 0.349, *p* = .016).^14^ In advanced adenocarcinomas with PD-L1 expression ≥50% treated with first-line pembrolizumab, *KRAS*m NSCLC (*n* = 62) showed a significant PFS benefit compared to *KRAS*wt (*n* = 57) (13.3 vs. 6.2 months, *p* = .05), with no significant difference in OS (23.4 vs. 26.1 months, *p* = .74).^20^ A retrospective study by Li et al. demonstrated that first-line pembrolizumab in combination with carboplatin, paclitaxel (for squamous cancers), or pemetrexed (for non-squamous cancers) for the treatment of *KRAS*m NSCLC (*n* = 23) had a higher PFS (12.8 vs. 9.7 months, *p* < .05) and OS (21.4 vs. 26.1 months, *p* = .74) than *KRAS*wt (*n* = 57), with *KRAS* mutation as a favorable factor for prolongation of OS (HR = 2.552, 95% confidence interval (CI): 1.141–5.708; *p* = .023).^21^

In addition, a retrospective study of long-term responders (LTRs) to first-line immunotherapy in NSCLC showed that *KRAS* mutations were more common in LTRs than in non-responders (39.4% vs. 28%, *n* = 13 vs. 7, *p* = .366).^42^ Similarly, a study by Notario et al. showed the enrichment of *KRAS* G12C mutations in LTRs (64%, *p* = .09).^43^ The response and efficacy of *KRAS*m NSCLC cells to immunotherapy may be related to the immune microenvironment. A retrospective study by Liu et al., which did not limit the number of lines of immune checkpoint inhibitor (ICI) treatment, showed that an increased tumor mutational burden (TMB) was associated with increased immunogenicity and that *KRAS*m NSCLC responded better to ICIs.^44^

However, other studies have suggested no significant difference between the efficacy of first-line immunotherapy for advanced *KRAS*m and *KRAS*wt NSCLC, either as ICI monotherapy or as immunochemotherapy. In terms of ICI monotherapy, the analysis of Justeau et al. based on a multicenter retrospective study (ESCKEYP) showed that among patients with non-squamous advanced NSCLC with PD-L1 ≥50% treated with first-line pembrolizumab, the *KRAS* G12C mutation group (*n* = 86), the *KRAS* non-G12C mutation group (*n* = 141), and the KRAS wild-type group (*n* = 454) were not significantly different regarding PFS (7 vs. 4.8 vs. 8.5 months, *p* = .2284) and OS (18.4 vs. 20.6 vs. 27.1 months, *p* = .5664).^22^ In addition, a retrospective study by Noordhof et al. showed that in patients with stage IV adenocarcinoma with PD-L1 expression ≥50% treated with first-line pembrolizumab, OS (19.2 vs. 16.8 months, *p* = .86) was not significantly different.^23^ Similarly, a study by Kartolo et al. demonstrated that advanced NSCLC with PD-L1 expression ≥50% treated with first-line anti-PD-1/PD-L1 monotherapy showed no significant difference in the ORR (37% vs. 26%, *p* = .268), PFS (6.0 vs. 5.4 months, *p* = .416), OS (12.9 vs. 19.3 months, *p* = .87) between the *KRAS*m group (*n* = 30) and the *KRAS*wt group (*n* = 29).^24^ In terms of immunochemotherapy, a KEYNOTE-189-based analysis by Gadgeel et al. showed that 59 cases of *KRAS*m NSCLC treated with first-line pembrolizumab combined with chemotherapy had a similar ORR (40.7% vs. 47.6%), PFS (9 vs. 9 months), and OS (21 vs. 23 months) compared to 145 cases of *KRAS*wt NSCLC.^25^ A study by Alessi et al. also found no significant differences in ORR, PFS, or OS between first-line immunochemotherapy in the *KRAS*m group (*n* = 351) and the *KRAS*wt group (*n* = 526) in advanced non-squamous NSCLC.^27^ Other studies that did not differentiate between ICI monotherapy and immunochemotherapy also suggested that the outcomes of first-line immunotherapy for *KRAS*m and *KRAS*wt NSCLC were not significantly different. Sun et al. showed that although OS in 363 cases of *KRAS*m NSCLC treated with first-line immunologic monotherapy was significantly longer than in 342 *KRAS*wt NSCLC (21.1 vs. 13.6 months, *p* = .03), OS in 210 *KRAS*m NSCLC treated with first-line immune-combination chemotherapy was not significantly different from that in 212 cases of *KRAS*wt NSCLC (20.0 vs 19.3 months, *p* = .93).^19^ A study by Veccia et al. showed no significant difference in OS (14.7 vs. 14.9 months, *p* = .529) between *KRAS*m (*n* = 50) and *KRAS*wt (*n* = 69) NSCLC treated with first-line immunochemotherapy or ICI monotherapy.^28^

Therefore, the results of several studies suggest that the efficacy of first-line immunotherapy for *KRAS*m NSCLC may be superior or at least comparable to that for *KRAS*wt NSCLC, especially in patients with positive PD-L1 expression.

### 4.2. ICI monotherapy improves treatment outcomes in KRASm NSCLC

First-line ICI monotherapy improves treatment outcomes in patients with *KRAS*m NSCLC compared to chemotherapy. In the analysis by Mok et al., based on the KEYNOTE-042 trial, 30 cases of *KRAS*m and 127 cases of *KRAS*wt advanced NSCLC with PD-L1 expression ≥1% were treated with first-line pembrolizumab.^29^ Patients with *KRAS*m showed significantly improved ORR (56.7% vs. 18%), PFS (12.3 vs. 6.2 months, HR = 0.51), and OS (28.4 vs. 11.0 months, HR = 0.42) compared to chemotherapy.^29^ Another single-center retrospective study by Liu et al. also showed that first-line single-agent immunotherapy (*n* = 50) significantly improved the ORR (44.00% vs. 30.43%), DCR (96.00% vs. 80.00%), PFS (11.7 vs. 7.0 months, *p* < .001), and OS (28.4 vs. 11.0 months, HR = 0.42) in *KRAS*m NSCLC compared to chemotherapy (*n* = 115).^30^ Further subgroup analyses showed that in NSCLC with PD-L1 expression ≥1%, first-line immunotherapy was associated with a significantly higher PFS (12.9 vs. 9.0 months, *p* = .011) and a significantly lower risk of disease progression (HR = 0.377, *p* = .020) compared to chemotherapy.^30^

In patients with *KRAS*m NSCLC with PD-L1 expression ≥50%, ICI monotherapy could serve as the first-line therapy. A real-world study by Velcheti et al. of advanced NSCLC with PD-L1 expression ≥50% treated with first-line pembrolizumab demonstrated that the median real-world time on treatment (rwToT) for first-line pembrolizumab in 164 patients with *KRAS*m with ECOG PS scores of 1–2 was 7.6 months (95% CI: 6.3–10.6 months), and the median rwToT for 166 patients with *KRAS*wt was 7.0 months (95% CI: 5.3–9.3 months).^45^ Thus, first-line ICI monotherapy showed a survival benefit in patients with PD-L1-overexpressing NSCLC, with or without *KRAS* mutations.

Therefore, first-line ICI monotherapy improves treatment outcomes in *KRAS*m NSCLC compared to chemotherapy, especially in patients with PD-L1 expression ≥50%.

### 4.3. Immunochemotherapy is more effective than other treatments in KRASm NSCLC

First-line immunochemotherapy was more effective than chemotherapy in treating *KRAS*m NSCLC. An analysis based on KEYNOTE-189 by Gadgeel et al. showed that in treating *KRAS*m advanced NSCLC, first-line pembrolizumab in combination with platinum-containing chemotherapy (*n* = 59) had a significantly higher ORR (40.7% vs. 26.7%) and a trend toward a prolonged PFS (9 vs. 5 months; HR = 0.47, 95% CI: 0.29–0.77) and OS (21 vs. 14 months; HR = 0.79, 95% CI: 0.45–1.38) compared with the chemotherapy group (*n* = 30).^25^ Similar results have been obtained in multiple retrospective studies. A study by Gu et al. showed that first-line ICI combined with platinum-containing chemotherapy (*n* = 33) in *KRAS*m NSCLC significantly increased PFS (7.4 vs. 4.5 months, *p* = .035) and OS (24.1 vs. 13.2 months, *p* = .007) compared to platinum-containing chemotherapy (*n* = 37).^31^ A retrospective study by Wang et al. also demonstrated that the OS (17 vs. 12 months, *p* = .11) was longer in the first-line immunochemotherapy group (*n* = 11) than in the non-immunotherapy group (*n* = 10, including chemotherapy and antivascular therapy) for *KRAS*m NSCLC, but the difference was not significant.^32^ Similarly, a pooled analysis by Nakajima et al. showed that for first-line treatment of *KRAS*m NSCLC, immunochemotherapy (*n* = 219) had ORR (46% vs. 35%) and OS (22.4 vs. 17.1 months) benefits over chemotherapy (*n* = 201).^26^

Compared to chemotherapy combined with antivascular therapy, *KRAS*m NSCLC was better treated with first-line immunochemotherapy. Sun et al. showed that in advanced *KRAS*m NSCLC (*n* = 76), first-line immunochemotherapy had a significant benefit in terms of ORR (47.4% vs. 31.1% vs. 21.2%), PFS (16.9 vs. 4.6 vs. 7.0 months), and OS (37.1 vs. 19.8 vs. 20.7 months) over chemotherapy (*n* = 74) or chemotherapy combined with antivascular therapy (*n* = 33).^33^

*KRAS*m NSCLC was treated more effectively with first-line immunochemotherapy than with ICIs alone. Nakajima et al. demonstrated that compared with first-line ICIs alone, *KRAS*m NSCLC with first-line immunochemotherapy (*n* = 219) resulted in an improved ORR (46% vs. 37%) and OS (22.4 vs. 16.2 months).^26^ However, in patients with *KRAS*m NSCLC and high PD-L1 expression, first-line immunochemotherapy did not show an additional survival benefit compared with ICI monotherapy. Sun et al. demonstrated that among 573 patients with *KRAS*m NSCLC and PD-L1 expression ≥50%, first-line immunochemotherapy (*n* = 210) and ICI monotherapy (*n* = 363) showed no significant differences in OS (20.0 vs. 21.1 months, *p* = .78).^19^

Thus, first-line immunochemotherapy is superior to other therapies, such as chemotherapy, chemotherapy combined with antivascular therapy, and ICI monotherapy, in patients with *KRAS*m NSCLC. In patients with high PD-L1 expression, the efficacy of immunochemotherapy is similar to that of ICI monotherapy.

### 4.4. Immunotherapy combined with chemotherapy and antivascular therapy improves outcomes in the first-line treatment of KRASm NSCLC

Combining antivascular therapy with immunochemotherapy improves the outcomes of first-line treatment for *KRAS*m NSCLC. Based on the IMpower150 trial, West et al. showed that in *KRAS*m non-squamous NSCLC, first-line atezolizumab/bevacizumab/carboplatin/paclitaxel (ABCP) was more effective in prolonging PFS (8.1 vs. 5.8 vs. 4.8 months) and OS (19.8 vs. 9.9 vs. 11.7 months) compared to either the bevacizumab/carboplatin/paclitaxel (BCP) regimen (*n* = 71) or the atezolizumab/carboplatin/paclitaxel (ACP) regimen (*n* = 74).^34^ The ABCP regimen improved the OS (HR = 0.50; 95% CI: 0.34–0.72 vs. HR = 0.63; 95% CI: 0.43–0.91) and PFS (HR = 0.42; 95% CI: 0.29–0.61 vs. HR = 0.80; 95% CI: 0.56–1.80) more significantly than the ACP regimen. Thus, immunotherapy combined with chemotherapy and antivascular therapy may further improve the first-line treatment outcomes.

## 5. Impact of KRAS mutant subtypes on the efficacy of immunotherapy

In advanced *KRAS*m NSCLC, G12C is the most common mutated subtype. Several studies have shown that first-line immunotherapy is more effective for patients with *KRAS* G12C mutations than for those with other *KRAS* mutations. For first-line immunotherapy in combination with chemotherapy, Elkrief et al. showed a significant PFS (6.8 vs. 5.4 months, *p* = .006) and OS (15 vs. 12 months, *p* = .12) benefit in the *KRAS* G12C group (*n* = 138) over the non-G12C group (*n* = 185).^46^ Cefalì et al. demonstrated that 11 of 44 patients with *KRAS*m NSCLC with PD-L1 expression ≥50% treated with first-line ICI showed a significantly longer PFS in the *KRAS* G12C than in the non-G12C group (14.6 vs. 6.5 months, *p* = .03).^47^ Similarly, NSCLC with PD-L1 expression ≥50% treated with first-line pembrolizumab showed significant benefits in ORR (63.3% vs. 36.0%, *p* = .05) and PFS (19.8 vs. 5.8 months, *p* = .001) in 32 cases in the *KRAS* G12C group than in the non-G12C group, with a non-significant trend toward a longer OS (HR = 0.50, 95% CI: 0.25–1.01, *p* = .06).^20^ Attili et al. showed that for stage IV non-squamous NSCLC with PD-L1 expression <50%, the G12C mutation was significantly associated with PFS benefits with first-line immunochemotherapy (HR = 0.29, 95% CI: 0.10–0.91).^48^

However, other studies have shown no significant difference in the efficacy of first-line immunotherapy between *KRAS* G12C mutations and non-G12C mutations. Justeau et al. demonstrated that in advanced NSCLC patients with PD-L1 expression ≥50% treated with first-line pembrolizumab, the *KRAS* G12C mutation group (*n* = 86) and the *KRAS* non-G12C mutation group (*n* = 141) showed no significant difference in ORR (47% vs. 40%), PFS (7 vs. 4.8 months), and OS (18.4 vs. 20.6 months).^22^ A retrospective study by Arbour et al. showed that in NSCLC receiving first-line immunotherapy, patients with the *KRAS* G12C mutation (*n* = 352) had comparable PFS (3.7 vs. 3.3 months, *p* = .89) to the non-G12C mutation group (*n* = 418).^49^

The relationship between *KRAS* mutant subtypes and immunotherapy efficacy may be associated with PD-L1 expression levels. Several studies have suggested that the G12D mutation is associated with low PD-L1 expression, whereas the G12C mutation is associated with high PD-L1 expression.^50,51^ In vitro experiments suggested that ICIs combined with paclitaxel can recruit CD8+ tumor-infiltrating lymphocytes (TILs) by increasing CXCL10/CXCL11 levels and can inhibit tumor growth more effectively than ICIs alone in tumors with *KRAS* G12D mutations, suggesting that patients with *KRAS* G12D mutations can be treated with first-line ICI combined with paclitaxel therapy.^51^ For G12C, an analysis based on clinical genomic data from 10,023 patients with NSCLC showed that *KRAS* G12C-mutated NSCLC was associated with a high TMB and PD-L1 expression ≥50%.^8,52^

In summary, first-line immunotherapy for *KRAS* G12C mutations may have a better prognosis than that for other mutation subtypes, which may be related to PD-L1 expression levels.

## 6. Concurrent mutations are associated with the prognosis of KRASm NSCLC

Concurrent mutations in *STK11*, *KEAP1*, and *TP53* were associated with the prognosis of *KRAS*-mutant NSCLC, and had implications on first-line treatment strategies.

The *STK11* co-mutation was consistently associated with poor prognosis in *KRAS*m NSCLC. Several retrospective studies showed that patients with *KRAS* and *STK11* mutations have shorter overall survival (OS) and progression-free survival (PFS) than those without these co-mutations.^53–56^ Similarly, the *KEAP1* co-mutation was also related to worse PFS and OS in KRASm NSCLC patients.^34,54,55,57^ The adverse prognostic roles of *STK11* and *KEAP1* co-mutations might be related to lower PD-L1 expression. Analysis of Negrao et al. suggested that negative PD-L1 expression (PD-L1 < 1%) was more common in *KRAS*m patients with the *STK11* or *KEAP1* mutation and was related to decreased PFS and OS.^58^

On the other hand, the role of *TP53* co-mutation in *KRAS*m NSCLC was more complex. While some research posed that *TP53* co-mutations were not related to survival outcomes, others suggested that *TP53* co-mutations were related to survival benefits of KRASm NSCLC, especially in first-line immunotherapy.^21,27,55,57^ Additionally, Aredo et al. showed that *TP53* co-mutations were more frequently found with high PD-L1 expression (≥50%).^53^ This association may explain the prolonged survival in *KRAS*m patients with *TP53* co-mutations receiving immunotherapy.

Co-mutations with *STK11*, *KEAP1* and *TP53* had substantial implications on first-line treatment strategies for *KRAS*m NSCLC. For *STK11* or *KEAP1* co-mutations, first-line treatment options primarily include chemoimmunotherapy or the combination of chemotherapy and antivascular therapy. West et al. showed that first-line ABCP regimens had significant PFS (6.0 vs. 3.2 vs. 3.4 months) and OS (11.1 vs. 7.9 vs. 8.7 months) benefits over ACP or BCP regimens in *KRAS*m NSCLC with *STK11* or *KEAP1* co-mutations.^34^ However, Sun et al. suggested that *KRAS*m/*STK11*m NSCLC patients with first-line chemotherapy combined with bevacizumab had a PFS benefit (7.0 vs. 4.4 vs. 3.9 months, *p* = .043) compared with chemoimmunotherapy and chemotherapy groups.^33^

On the other hand, for *KRAS*m NSCLC with *TP53* co-mutation, first-line chemoimmunotherapy should be considered. Analyzing the IMpower 150 trial showed that PFS (14.3 vs. 4.6 vs. 4.2 months) and OS (30.6 vs. 11.7 vs. 9.5 months) in the first-line ABCP regimen were significantly longer than in ACP or BCP regimens.^34^ Consistently, research by Sun et al. indicated that PFS (18.7 vs. 6.1 vs. 6.8 months, *p* < .0001) of *KRAS*m NSCLC with *TP53* mutation were significantly longer in ICI combined with chemotherapy than those in the chemotherapy alone or chemotherapy combined with antivascular therapy.^33^

Therefore, concurrent mutations of *STK11* or *KEAP1* were negative prognostic factors for *KRAS*m NSCLC, while *TP53* seemed to be associated with improved survival outcomes. Combining immunotherapy and chemotherapy enhanced the outcomes in *KRAS*-mutant NSCLC patients with these concurrent mutations and could be applied in first-line treatment.

## 7. Kras-targeting therapies

Various new therapeutic approaches are available as first-line candidates for treating NSCLC with *KRAS* mutations. These therapies include *KRAS*-targeting therapies, metabolic therapies, and their combinations with existing first-line agents. Currently, these therapies are still being evaluated in clinical trials as second-line or higher treatment options.

### 7.1. KRAS(OFF) inhibitors

Drugs targeting the switch region of the KRAS G12C protein, including sotorasib (AMG 510) and adagrasib (MRTX849), have been developed (Table 2). Sotorasib (AMG 510), the first KRAS G12C inhibitor, binds to a cysteine residue in the switch II region and prevents activation of KRAS.^59^ Based on the results of phase 1 and single-arm phase 2 trials, sotorasib was first approved by the Food and Drug Administration (FDA) in 2021 for the treatment of advanced NSCLC in the second line and beyond.^60–62^ In the phase 3 trial, sotorasib had a significantly longer PFS than docetaxel (5.6 vs. 4.5 months, HR = 0.66, *p* = .0017), with fewer grade 3 or 4 adverse events.^63^ A first-line trial of sotorasib (NCT04933695) is currently underway. Thus, there is growing evidence that sotorasib is a promising candidate for treating NSCLC with *KRAS* G12C mutation.

**Table 2.** Ongoing clinical trials for sotorasib and adagrasib in KRAS mutant NSCLC.

Treatment	Regimen	Trial number
**Sotorasib**		
Sotorasib monotherapy	Sotorasib	NCT03600883, NCT03600883, NCT04303780, NCT04625647, NCT05398094, NCT04933695, NCT05451056, NCT05273047, NCT05311709, NCT06127940, NCT06333678, NCT05400577, NCT05631249
Sotorasib + Chemotherapy	Sotorasib + Platinum doublet	NCT05920356, NCT05118854
Sotorasib + Antivascular therapy	Sotorasib + MVASI	NCT05180422
Sotorasib + Aurora A kinase inhibitor	Sotorasib + VIC-1911	NCT05374538
Sotorasib + CXCL-8 inhibitor	Sotorasib + Ladarixin	NCT05815173, NCT05815186
Sotorasib + Tyrosine kinase inhibitor	Sotorasib + Lenvatinib/Tarloxotinib	NCT06068153
Sotorasib + HER2 inhibitor	Sotorasib + Tarloxotinib	NCT05313009
Sotorasib + RAF/MEK inhibitor	Sotorasib + Avutometinib + (Defactinib)	NCT05074810
Sotorasib + SHP2 inhibitor	Sotorasib + RMC-4630/BBP-398	NCT05054725, NCT05480865
Sotorasib + Proteasome inhibitor	Sotorasib + Carfilzomib	NCT06249282
**Adagrasib**		
Adagrasib monotherapy	Adagrasib	NCT03785249, NCT04685135, NCT05853575, NCT05673187
Adagrasib + Immunotherapy	Adagrasib + Pembrolizumab/Nivolumab	NCT04613596, NCT05472623
Adagrasib + RAF/MEK inhibitor	Adagrasib + Avutometinib	NCT05375994
Adagrasib + mTOR inhibitor	Adagrasib + Nab-Sirolimus	NCT05840510

Adagrasib (MRTX8499) is a recently developed KRAS G12C inhibitor. Based on a phase I/IB study, NSCLC patients with *KRAS* G12C mutations treated with adagrasib had a median PFS of 11.1 months.^64^ In 2022, adagrasib was approved for patients with advanced NSCLC with *KRAS* G12C mutations who had previously received systemic therapy.^65^ In the phase 2 study, among 112 patients for whom baseline disease assessment was available, the ORR was 42.9%, the median PFS was 6.5 months, the median OS was 12.6 months, and the incidence of treatment-related adverse events of grade 3 or higher was 44.8%.^66^ Preliminary results from the phase 1/2 KRYSTAL-12 trial (NCT04685135) showed that in patients with *KRAS* G12C-mutated NSCLC who had previously received chemotherapy or immunotherapy, after 9.4 months of follow-up, the adagrasib group showed significant benefit in ORR (31.9% vs. 9.2%, *p* < .0001) and PFS (5.49 vs. 3.84 months, *p* < .0001) compared to the docetaxel group, with a similar incidence of grade 3 and higher TRAE (47.0% vs 45.7%).^67^ Further clinical trials are ongoing to explore the efficacy and safety of adagrasib monotherapy and combination therapy in advanced NSCLC.

Combination therapies for other KRAS inhibitors have also shown better efficacy. For combining immunotherapy, preliminary results from the phase 1/2 LOXO-RAS-20001 trial (NCT04956640) of the second-generation KRAS G12C inhibitor, olomorasib (LY3537982), in combination with pembrolizumab to treat advanced NSCLC, demonstrated an ORR of 63% in 50 patients at 6-month follow-up (95% CI: 44–80%), suggesting that KRAS inhibitors in combination with ICI may have superior efficacy.^68^ For combination chemotherapy, in the phase Ib CodeBreaK 101 (NCT04185883) study, 58 patients treated with first-line sotorasib in combination with pemetrexed and carboplatin had an ORR of 65% (95% CI: 46.5–80.3) and a median PFS of 10.8 months (95% CI: 5.4-NE months), with 30 patients (52%) experiencing grade 3–4 TRAE and 1 patient death.^69^ In terms of combining other targeted therapies, 27 patients with advanced NSCLC receiving the first-line KRAS G12C inhibitor fulzerasib (GFH925) in combination with the EGFR inhibitor cetuximab had an ORR of 80.0% (95% CI: 56.3–94.3%) in the phase II KROCUS study (NCT05756153), with a DCR of 100% (95% CI: 83.2–100.0%), with 5 patients experiencing grade 3 or higher TRAE.^70^ Thus, combination therapies with KRAS inhibitors are expected to be a future first-line treatment option.

Several ongoing trials are evaluating the outcomes of sotorasib and adagrasib monotherapies and in combination with other therapies (Table 2).

### 7.2. KRAS(ON) inhibitors

Sotorasib and adagrasib are classified as KRAS(OFF) inhibitors, targeting the KRAS protein in its inactive state. In contrast, KRAS(ON) inhibitors specifically target the active, GTP-bound KRAS to combat KRAS-driven cancers.^71^ Several KRAS(ON) inhibitors have been developed and tested in preclinical and clinical studies, including RMC-6236, RMC-4998, and RMC-7977.

RMC-6236 combined cyclophilin A (CYPA) to target KRAS in an active state, forming a tri-complex that inhibited downstream signal transduction.^72^ Preclinical results suggested that RMC-6236 down-regulated RAS signaling, leading to tumor regression in the mouse xenograft model.^73^ Clinical trials are ongoing to evaluate RMC-6236 as a monotherapy and in combination with immune checkpoint inhibitors (NCT05379985, NCT06162221). Similarly, tri-complex inhibitor RMC-4998 could overcome resistance to sotorasib both in vitro and in vivo.^74^ Combining RMC-4998 and sotorasib inhibited cell proliferation and downstream signal transduction with increased efficacy on *KRAS*-mutant tumors.^74^ Currently, a phase 1/2 clinical trial is ongoing to test RMC-6291 in *KRAS*-mutant tumors (NCT05462717). In addition, RMC-7977 was a tri-complex RAS inhibitor targeting KRAS, NRAS, and HRAS. In preclinical studies, RMC-7977 led to the regression of *KRAS*-mutant tumors and showed substantial efficacy in tumor models with *KRAS* exon 12 alterations.^75^ Recently, RMC-7977 has been assessed in patients with *KRAS*-mutant solid tumors (NCT05379985).

Further research was needed to evaluate the efficacy and safety of KRAS(ON) inhibitors before application in the clinic.

In addition to the drugs mentioned above, several KRAS G12C inhibitors (LY3499446, GDC-6036, D-1553, JDQ443, BI 1,823,911, LY3537982, JAB-21822, YL-15293, and RMC-6291) and KRAS G12D inhibitors (KRpep-2d, KS-58, and MRTX1133) have been used in preclinical and clinical trials.^76–79^ Further evidence is needed for KRAS-targeted therapies as the first-line treatment of *KRAS*-mutant NSCLC.

## 8. Conclusion and future perspectives

*KRAS* mutations are common in NSCLC. Clinical evidence has shown that first-line chemotherapy or chemotherapy combined with antivascular therapy for *KRAS*m NSCLC is not as effective as for *KRAS*wt NSCLC, but first-line immunotherapy is better than or at least comparable to *KRAS*wt NSCLC. Chemotherapy combined with immunotherapy is the preferred first-line treatment for *KRAS*m NSCLC, with better efficacy when combined with antivascular therapy. ICI monotherapy was also an option for patients with a PD-L1 tumor proportion score ≥50%. In addition to chemotherapy, antivascular therapies, and immunotherapy, a variety of emerging treatments are expected to become first-line therapies in the future, and KRAS inhibitors, such as sotorasib and adagrasib, may gradually become first-line treatments for *KRAS*m NSCLC.

## Data Availability

Data sharing is not applicable to this article as no new data were created or analyzed in this study.
